# Genomic Analysis Reveals Diversified and Stress-Responsive Transport Repertoire in *Candidozyma (Candida) auris*

**DOI:** 10.3390/jof12030174

**Published:** 2026-02-28

**Authors:** Raymond Cai, Jianying Gu

**Affiliations:** 1 Keystone School, San Antonio, TX 78212, USA; raymondcai521@gmail.com; 2 Department of Biology, College of Staten Island, City University of New York, Staten Island, NY 10314, USA

**Keywords:** *Candidozyma auris*, transporter, drug resistance, genomics, metabolism, data mining

## Abstract

*Candidozyma (Candida) auris* is a fungal pathogen associated with life-threatening invasive infections and high mortality rates. It is becoming a major global public health concern due to its ability to resist multiple antifungal drugs and spread in healthcare settings. Despite this, little is known about the mechanisms underlying drug resistance, fungal development, pathogenesis, and virulence. Among the factors contributing to these processes, transporters play a central role in fungal biology, regulating nutrient acquisition, metabolite exchange, ion homeostasis, and drug efflux. However, the composition and diversity of transporter systems in *C. auris* remain poorly defined. Through genomic analysis, we identified 686 transporters and 125 accessory factors involved in transport in *C. auris*, most of which had not been characterized. These transporters and accessory factors were classified into seven classes, 22 subclasses, and 215 families, reflecting substantial functional diversity. Comparative analyses with other pathogenic *Candida* species and *Saccharomyces cerevisiae* reveal lineage-specific divergence in several transporter families. We also integrated multiple publicly available RNA-seq datasets encompassing antifungal drug exposure and drug-resistant isolates and identified subsets of transporters that are transcriptionally responsive in distinct antifungal conditions, including members of families implicated in drug transport, metabolism, and ion homeostasis. Together, this study defines the landscape of transporter systems in *C. auris* and highlights transporter families that may contribute to stress adaptation and antifungal responses, providing a resource for future functional and mechanistic investigations.

## 1. Introduction

*Candidozyma (Candida) auris* is a fungal pathogen that infects multiple organs and has been associated with mortality rates ranging from 30% to 60% [1,2]. In 2022, the World Health Organization listed *C. auris* as a “critical priority” pathogen due to its rapid global spread and resistance to multiple antifungal drugs [3]. In hospitals and long-term care settings, where the fungus can spread via contact with patients or contaminated medical equipment, *C. auris* infections have increased significantly among high-risk populations, including elderly and immunocompromised individuals [4,5,6]. Following the COVID-19 pandemic, both the frequency and mortality of *C. auris* infections increased, likely driven by the rise in immunocompromised patients and the widespread use of antibiotics and invasive medical devices such as central venous catheters [7,8]. Understanding the molecular basis of its survival, pathogenicity, and drug resistance is therefore an urgent priority in medical mycology.

Transporters, which move diverse molecules across cellular membranes, are central to fungal metabolism, physiology, virulence, and drug resistance. In pathogenic fungi, they are not merely conduits for nutrients, but also contribute to survival within the host by maintaining ion balance, exporting antifungal compounds, and mediating interactions with host environments [9,10]. Specific transporter families, such as the ATP-binding cassette (ABC) and major facilitator superfamily (MFS), are directly implicated in antifungal resistance by acting as efflux pumps that expel drugs from the cell [11,12]. In *C. auris*, clinical resistance to azoles and other drugs has been linked to these transporter families [13,14,15,16]. Despite this, a comprehensive overview of the transporter repertoire encoded in the *C. auris* genome remains lacking.

Phylogenetically, *C. auris* belongs to the subphylum Saccharomycotina [17]. In this study, *Saccharomyces cerevisiae*, a model organism within this subphylum, serves as a functional baseline due to its extensively curated, experimentally validated proteome, providing a reference framework for conserved transporter families.

While *C. auris* is recognized as a nosocomial pathogen, its emergence may stem from ecological adaptations acquired in its natural reservoirs. The recent isolation of *C. auris* from mangroves and salt marshes in the Andaman Islands provides evidence of its existence in a natural habitat [18]. Transitioning from hypersaline environments to the human host requires effective osmoregulation and ionic balancing, functions supported by the membrane transport system. Moreover, the ‘global warming hypothesis’ posits that *C. auris* developed thermotolerance in natural reservoirs, likely facilitated by the expansion of stress-responsive transporter families [19]. Characterizing these ecological origins helps explain the selection of transport systems that confer both environmental resilience and clinical multidrug resistance, providing the machinery necessary to navigate nutrient scarcity and antifungal pressure within the host.

With complete genome sequences available [20,21], genome-wide identification and classification of transporters can be performed using curated resources such as the Transporter Classification Database (TCDB) [22], which organizes transport systems into evolutionarily and functionally defined families. A systematic analysis of the *C. auris* transporter repertoire is therefore feasible and may provide insights into both intrinsic physiology and antifungal response mechanisms.

In this study, we report the identification of 686 putative transporters and 125 accessory factors involved in transport in the genome of the *C. auris* B8441 strain. These transporters are predicted to mediate a broad spectrum of cellular processes, including metabolite exchange, nutrient acquisition, detoxification, ion regulation, and signal transduction. This work provides an initial framework for exploring the organization and functional roles of the transport systems in *C. auris*, a rising pathogen whose molecular transport processes remain incompletely characterized.

## 2. Materials and Methods

### 2.1. Sequence Data Retrieval

**Figure 1:**
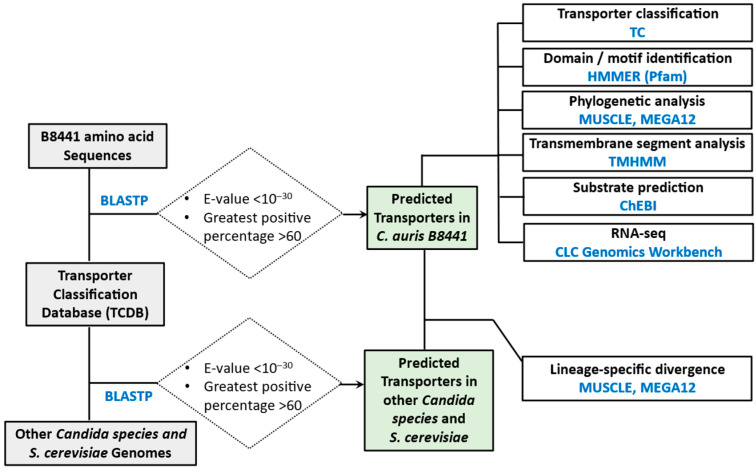
Overview of the workflow used for genomic identification and characterization of *C. aruis* transporters.

The workflow of our genomic analysis is summarized in Figure 1. The translated amino acid sequences of the reference genome, the *C. auris* B8441 strain [20], were retrieved from the *Candida* Genome Database (http://www.candidagenome.org/, accessed on 29 August 2025) [23].

The Transporter Classification Database (TCDB) (https://www.tcdb.org/, accessed on 28 August 2025), a curated resource that compiles protein sequences and expert-reviewed information on transport systems across all domains of life, including their classification, structural features, evolutionary relationships, biological functions, and biomedical relevance, served as the reference framework for identifying and categorizing transporters in *C. auris* [22].

### 2.2. Transporter Repertoire Prediction and Classification in C. auris

All predicted protein sequences from the *C. auris* B8441 genome were queried against the TCDB using BLASTP v2.17.0 to identify putative transporters [24]. Candidate homologs were retained only if they met stringent criteria of an E-value ≤ 10^−30^ and ≥60% positive sequence similarity [25,26]. Functional annotation was assigned based on the best-scoring TCDB match, defined by the lowest E-value in conjunction with the highest degree of sequence similarity.

The Transport Classification (TC) system was used to classify predicted *C. auris* transporters [27]. It is a formal, internationally recognized classification and nomenclature system for membrane transport proteins. In the five-character TC numbering system (V.W.X.Y.Z), each position denotes a distinct level of classification, indicating the transporter’s class, subclass, family, subfamily, and the type of substrate it carries.

Structural features of the predicted *C. auris* transporters were predicted using Pfam, which identifies conserved domains and sequence motifs through profile Hidden Markov Models (HMMs) [28]. Membrane topology was assessed using the TMHMM algorithm, which predicts transmembrane segments (TMSs) and characterizes membrane-spanning regions [29]. In addition, putative substrate specificities were inferred using the Chemical Entities of Biological Interest (ChEBI) ontology, a curated resource for metabolites and small molecules [30].

### 2.3. Multiple Sequence Alignment and Phylogenetic Reconstruction

MUSCLE was employed to produce multiple sequence alignments [31,32]. Phylogenetic trees were reconstructed with MEGA12 [33] using the neighbor-joining [34] and the maximum-likelihood [35] methods. Support for individual branches was estimated through 1000 bootstrap resampling replicates [36].

### 2.4. Transcriptomic Analysis of Predicted Transporters in Drug-Resistant Isolates or Drug-Exposed Conditions

To evaluate the transcriptional regulation of predicted transporter genes, we reanalyzed three previously published RNA-seq datasets generated from *C. auris* under drug-resistant or antifungal-exposed conditions.

(1) Polyene Resistance: Shivarathri et al. (2022) [37] compared baseline transcriptomes of three amphotericin B (AmB)-resistant clinical isolates (AmBR1-3) against susceptible ones.
(2) Echinocandin Response: Zamith-Miranda et al. (2021) [38] exposed strains B8441 and MMC1 to caspofungin for 24 h to capture the immediate transcriptional response, enabling the identification of drug-induced transcriptional changes.
(3) Azole Adaptation: Bing et al. (2020) [39] tracked how *C. auris* adapts to long-term fluconazole pressure through experimental evolution. They used serial passaging in increasing drug concentrations to generate resistant lineages from a susceptible ancestor. We used their RNA-seq data from the parental strain and the evolved resistant descendants to assess resistance-associated transcriptional alterations.

Raw FASTQ files for each RNA-seq dataset were downloaded separately from the Gene Expression Omnibus (GEO) and the Sequence Read Archive (SRA) under accession numbers GSE190920, SRP295539, and GSE136768. Each dataset was processed and analyzed independently to avoid introducing batch effects arising from differences in experimental design, strain background, sequencing platform, or laboratory conditions. At no point were read counts, expression matrices, or normalization factors merged or jointly modeled across datasets. RNA-seq analyses were performed using CLC Genomics Workbench version 25 (Qiagen, Redwood City, CA, USA). For each dataset, low-quality reads and adapter sequences were removed, and trimmed reads were aligned to the *C. auris* B8441 reference genome. Transcript abundance was estimated using the expectation–maximization (EM) algorithm. Read counts were normalized within each dataset using the trimmed mean of M-values (TMM) method [40] implemented in edgeR [41,42]. Differential expression analysis was conducted separately for each dataset using a negative binomial generalized linear model (GLM) [42], followed by multiple testing correction with the Benjamini–Hochberg procedure [43] to control the false discovery rate (FDR). Genes with an FDR-adjusted *p*-value < 0.05 and an absolute log_2_ fold change ≥ 1 were considered differentially expressed.

### 2.5. Comparisons with Transporters in S. cerevisiae and Other Candida Species

Since most *Candida* species lack curated transporter annotations, we built a comparative framework using proteomes from *C. albicans* SC5314, *C. dubliniensis* CD36, *Nakaseomyces (Candida) glabrata* CBS138, *C. parapsilosis* CDC317, and *S. cerevisiae* S288C. *S. cerevisiae* was included as a well-annotated model yeast with an extensively characterized proteome, providing a functional and evolutionary reference framework.

Using the same stringent BLAST v2.17.0 cutoff criteria applied to *C. auris*, protein sequences from each species were queried against the Transporter Classification Database (TCDB) to identify putative transporter homologs. Identical thresholds for sequence similarity, coverage, and statistical significance were applied across all species to ensure comparability of transporter assignments.

To investigate evolutionary relationships and species-specific expansions, multiple sequence alignments and phylogenetic analyses were performed on the identified transporter families.

### 2.6. Phylogenetic Context of C. auris in the Subphylum Saccharomycotina

We selected 94 representative species across the subphylum Saccharomycotina, based on the phylogenetic framework of Opulente et al. [17]. Phylogenetic relationships were inferred from internal transcribed spacer (ITS) sequences of the ribosomal DNA. The ITS region is widely used as a universal fungal barcode because of its high variability across species. Multiple sequence alignment was performed with MUSCLE [31,32]. The maximum-likelihood [35] tree was reconstructed in MEGA12 [33], with clade robustness evaluated via 1000 bootstrap replicates [36].

## 3. Results

### 3.1. C. auris Possesses a Rich Repertoire of Transporters

To characterize the transporter repertoire of *C. auris*, translated amino acid sequences from the *C. auris* reference strain B8441 were systematically queried against the Transporter Classification Database (TCDB) [22]. To maximize specificity and reduce spurious assignments, a stringent filtering strategy was applied, retaining hits with an E-value ≤ 10^−30^ and a sequence similarity ≥ 60% at the amino acid level. We identified 686 transporters and 125 accessory factors involved in transport in *C. auris* (Table S1). Among these, 412 were functionally annotated and classified based on sequence homology to experimentally characterized transporters in *S. cerevisiae* (Table S1). Owing to its extensively curated transporters, *S. cerevisiae* served as the principal functional reference for assigning conserved transporter families.

**Figure 2:**
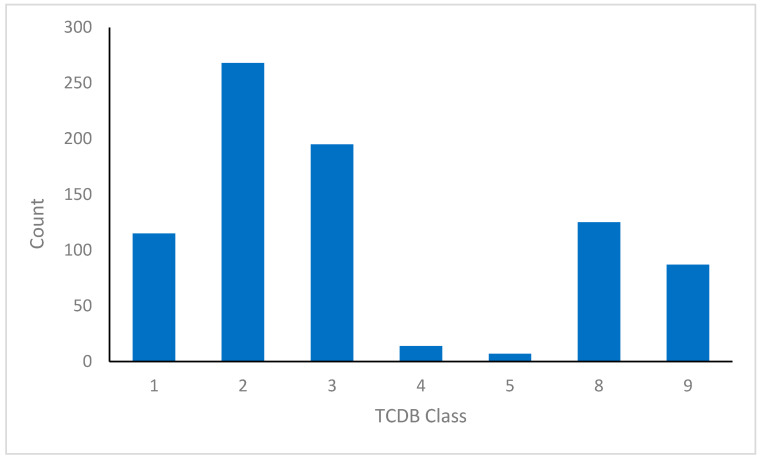
Classification of transporter families in *C. auris* genomes according to the Transporter Classification (TC) system. Categories include: Class 1, Channels/Pores; Class 2, Electrochemical Potential-Driven Transporters; Class 3, Primary Active Transporters; Class 4, Group Translocators; Class 5, Transmembrane Electron Carriers; Class 8, Accessory Factors Associated with Transport; and Class 9, Incompletely Characterized Transport Systems.

Utilizing domain specifications in conjunction with TC system nomenclature, the predicted transporters in *C. auris* were categorized into seven classes, 22 subclasses, and 215 families (Figure 2 and Table 1). Many of these predicted transporters were originally annotated as uncharacterized open reading frames (ORFs) in the initial genome sequencing project, reflecting the absence of functional assignment at the time. In the present study, they were assigned based on predicted structural attributes, transport mechanisms, and phylogenetic relatedness.

Electrochemical potential-driven transporters (Class 2) constituted the largest category in *C. auris*, representing over 33% of the predicted transporter complement Table S1). Over 98% (264 out of 268) of these proteins were annotated as porters, a diverse group of uniporters, antiporters, and symporters [44]. In fungal systems, transporters are essential for nutrient acquisition and detoxification of toxins and antifungal agents. The major facilitator superfamily (MFS) comprises 96 members. MFS transporters are of biological and clinical interest due to their well-documented roles in antifungal resistance across diverse pathogenic fungi, including *C. albicans* [45,46,47], *C. parapsilosis* [48], *N. glabratus* [49], *Penicillium digitatum* [50], *Aspergillus fumigatus* [51], and *Alternaria alternata* [52]. Further characterization of *C. auris* MFS transporters can be found in Section 3.2.2.

Primary active transporters (Class 3) formed the second largest group, comprising about 24% of the total transporter repertoire in *C. auris* (Table S1). By directly using ATP or alternative energy sources, these proteins transport solutes against their concentration gradients, contributing to nutrient acquisition and the export of various substrates. The ATP-binding cassette (ABC) family is particularly important in this class because ABC efflux pumps have been widely implicated in antifungal resistance across pathogenic fungi [26,53,54,55,56,57,58]. Twenty-six ABC transporters were detected in *C. auris*, and their phylogenetic and network characterizations are detailed in Section 3.2.1.

Class 1 transporters, which serve as pores and channels, made up approximately 14% of the transporter complement in *C. auris* and are widely conserved across the tree of life. Without an external energy source, these proteins mediate passive solute transport. The two subtypes that predominate in *C. auris* are α-type ion channels [59] and membrane-integrated channels [60].

Class 8 of the TC system included accessory factors that aid in the regulation, assembly, or stabilization of transporters rather than directly moving solutes. In *C. auris*, 125 such proteins were predicted. Their specific roles are not yet known, but in other fungi, Class 8 members have been linked to protein folding, membrane insertion, and the assembly of transporter complexes [61], suggesting similar functions in *C. auris*.

### 3.2. Transporters of Potential Biological and Clinical Importance in C. auris

A subset of the identified transporters may be crucial to the pathogenicity and biology of *C. auris*. Members of the ABC superfamily (TC 3.A.1) and the MFS (TC 2.A.1) are noteworthy because of their known roles in other fungi.

#### 3.2.1. ABC Transporters

The ABC transporters constitute one of the largest and most conserved protein families across all domains of life [62,63,64]. They contain nucleotide-binding domains (NBDs) and transmembrane domains (TMDs) [65,66]. ABC transporters mediate the movement of diverse substrates involved in nutrient uptake, ion homeostasis, and stress adaptation [67,68]. They are also well known for their role in multidrug resistance in fungi and other organisms [69,70,71]. Although ABC transporters have been extensively characterized in many fungal species [72], their diversity and functions in *C. auris* remain largely unexplored.

**Figure 3:**
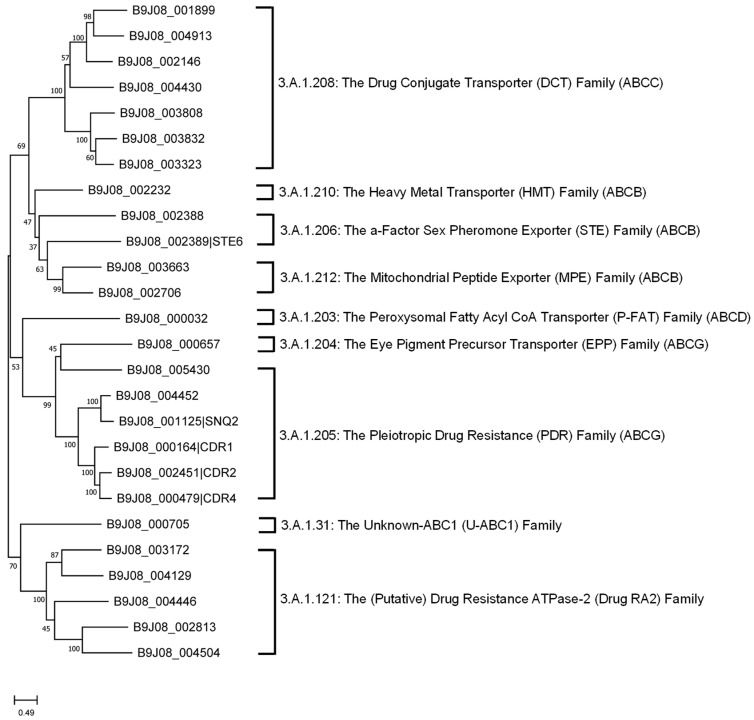
The phylogenetic tree of major ABC transporter families in *C. auris*. The phylogeny was inferred using the Maximum Likelihood method and the Jones-Taylor-Thornton model of amino acid substitutions [79], and the tree with the highest log likelihood (−68,636.59) is shown. The percentage of replicate trees in which the associated taxa clustered together (1000 replicates) is shown next to the branches [36]. The initial tree for the heuristic search was selected by choosing the tree with the superior log-likelihood between a Neighbor-Joining (NJ) tree [34] and a Maximum Parsimony (MP) tree [35]. Evolutionary analyses were conducted in MEGA12 [33].

We identified 26 putative ABC transporters in the *C. auris* strain B8441, all of which contain the canonical ABC domains. In comparison, 28 ABC transporters were previously predicted in the *C. auris* strain CBS 10913T [26]. Phylogenetic analysis grouped these proteins into nine families (Figure 3).

The Pleiotropic Drug Resistance (PDR) family (ABCG, TC 3.A.1.205) includes six homologs: *CDR1* (B9J08_000164), *CDR2* (B9J08_002451), *CDR4* (B9J08_000479), *SNQ2* (B9J08_001125), and two uncharacterized transporters (B9J08_004452 and B9J08_005430). Among these, *CDR1* encodes a major azole efflux pump; deletion of *CDR1* in clinical isolates significantly reduced azole resistance [16,73,74], making it a potential therapeutic target [13,75]. Notably, *CDR1* is a key downstream target of the transcription factor *TAC1B*, and activating mutations in *TAC1B* drive *CDR1* upregulation, thereby contributing to multidrug resistance phenotypes in *C. auris* [76,77]. The role of *CDR2* in *C. auris* remains unclear, though its ortholog in *C. albicans* contributes to fluconazole resistance [76]. *CDR4* and *SNQ2* are expressed at higher levels in multidrug-resistant *C. auris* isolates; however, their specific functions remain undefined [78].

The Drug Conjugate Transporter (DCT) family (ABCC, TC 3.A.1.208) comprises a broad group of ABC transporters that mediate the efflux of organic anions, toxins, drugs, and conjugated metabolites. In fungi, members of this family have been linked to resistance against antifungal agents, oxidative stress, and heavy metal toxicity [80,81,82,83]. In *C. auris*, seven putative ABCC-type transporters were identified (Figure 3), although their physiological and pharmacological roles remain unknown. Three genes (B9J08_003323, B9J08_003808, and B9J08_003832) share homology with *YOR1* from *S. cerevisiae*, which encodes an exporter of organic anions and confers resistance to oligomycin and tetracycline [80]. Another three genes (B9J08_001899, B9J08_002146, and B9J08_004913) are homologous to *S. cerevisiae YCF1*, a vacuolar transporter involved in sequestering heavy metals and glutathione conjugates to mitigate cellular stress [81,84]. Notably, two transporters, B9J08_002146 (ABCC) and B9J08_000677 (TC 2.A.59, Arsenical Resistance-3, *ACR3*), may contribute to arsenite resistance, as their *S. cerevisiae* homologs *YCF1* and *ACR3* are known to function cooperatively in arsenic detoxification pathways [85,86].

The ABCB group in *C. auris* includes three distinct families: (1) the a-Factor Sex Pheromone Exporter (STE) Family (TC 3.A.1.206), (2) the Mitochondrial Peptide Exporter (MPE) Family (TC 3.A.1.212), and (3) the Heavy Metal Transporter (HMT) Family (TC 3.A.1.210) (Figure 3). Although members of these families have been described in *S. cerevisiae* and other fungal species [72,87,88,89], their specific functions in *C. auris* have not yet been revealed.

Within the ABC superfamily of *C. auris*, five genes belong to the Putative Drug Resistance ATPase-2 (Drug RA2) Family (TC 3.A.1.121) in the ABCF group [90], and a gene is classified in the ABCD group, corresponding to the Peroxisomal Fatty Acyl-CoA Transporter (P-FAT) Family (TC 3.A.1.203), which may be involved in fatty acid metabolism and lipid biosynthesis [91].

#### 3.2.2. Major Facilitator Superfamily (MFS)

MFS (TC 2.A.1) comprises a broad class of membrane transport proteins that function as secondary carriers. Members of this superfamily are ubiquitous across bacteria, archaea, and eukaryotes, participating in the exchange of amino acids, sugars, drugs, and other small molecules essential for maintaining cellular homeostasis [92,93]. Beyond their physiological transport roles, MFS proteins are increasingly recognized for their contributions to antifungal resistance through the efflux process [9,12,94,95].

Like in other fungi, the MFS transporters in *C. auris* have undergone lineage-specific expansion. A total of 96 MFS members were identified, representing approximately 11% of the predicted transporter repertoire; most remain uncharacterized.

**Figure 4:**
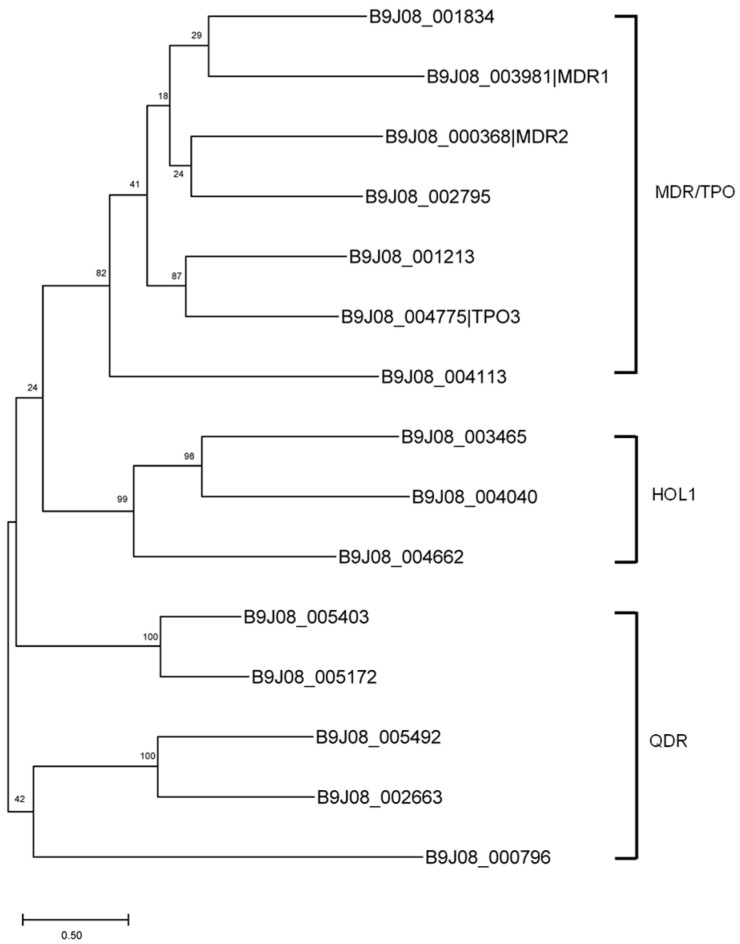
The phylogenetic tree of DHA1 transporters in *C. auris*. The phylogeny was inferred using the Maximum Likelihood method and Jones-Taylor-Thornton model [79] of amino acid substitutions and the tree with the highest log likelihood (−21,786.22) is shown. The percentage of replicate trees in which the associated taxa clustered together (1000 replicates) is shown next to the branches [36]. The initial tree for the heuristic search was selected by choosing the tree with the superior log-likelihood between a Neighbor-Joining (NJ) tree [34] and a Maximum Parsimony (MP) tree [96]. The NJ tree was constructed from a matrix of pairwise distances computed with the *p*-distance. The MP tree had the shortest length among 10 MP tree searches, each performed with a randomly generated starting tree. Evolutionary analyses were conducted in MEGA12 [33].

Among these, 15 belong to the Drug:H^+^ Antiporter-1 (DHA1) family (TC 2.A.1.2). Members of the DHA1 family have been extensively studied for their roles in drug tolerance, ion homeostasis, and virulence across diverse fungal species, including *S. cerevisiae*, *C. albicans*, *N. glabratus*, *C. dubliniensis*, *C. maltosa*, *A. fumigatus*, *Cryptococcus neoformans*, and *Fusarium graminearum* [47,49,97,98,99,100,101]. A previous genomic survey by Khatoon et al. identified 14 DHA1 transporters in *C. auris* [14]. Our analysis expanded this list by including two additional DHA1 transporters (B9J08_000796 and B9J08_003465) and excluding one gene (B9J08_002079) that did not meet the defined similarity criteria. Phylogenetic reconstruction revealed that these DHA1 transporters cluster into several functional clades (Figure 4). The MDR (multidrug resistance)/TPO clade includes seven homologs: *CauMDR1* (B9J08_003981), *CauMDR2* (B9J08_000368), *TPO3* (B9J08_004775), and four uncharacterized proteins. Notably, *CauMDR1* has been functionally characterized as an efflux pump that exports multiple antifungal agents [14]; the *TPO* (transporter of polyamines) transporter may contribute to maintaining the polyamine balance required for cell growth and differentiation [102].

The QDR (quinidine drug resistance) family comprises five members whose functions in *C. auris* remain to be elucidated; however, homologs in *C. albicans* have been implicated in biofilm formation and virulence [103]. In addition, *C. auris* encodes three *HOL1*-like transporters, whose counterparts in other fungi are involved in cation and histidinol transport [104].

**Figure 5:**
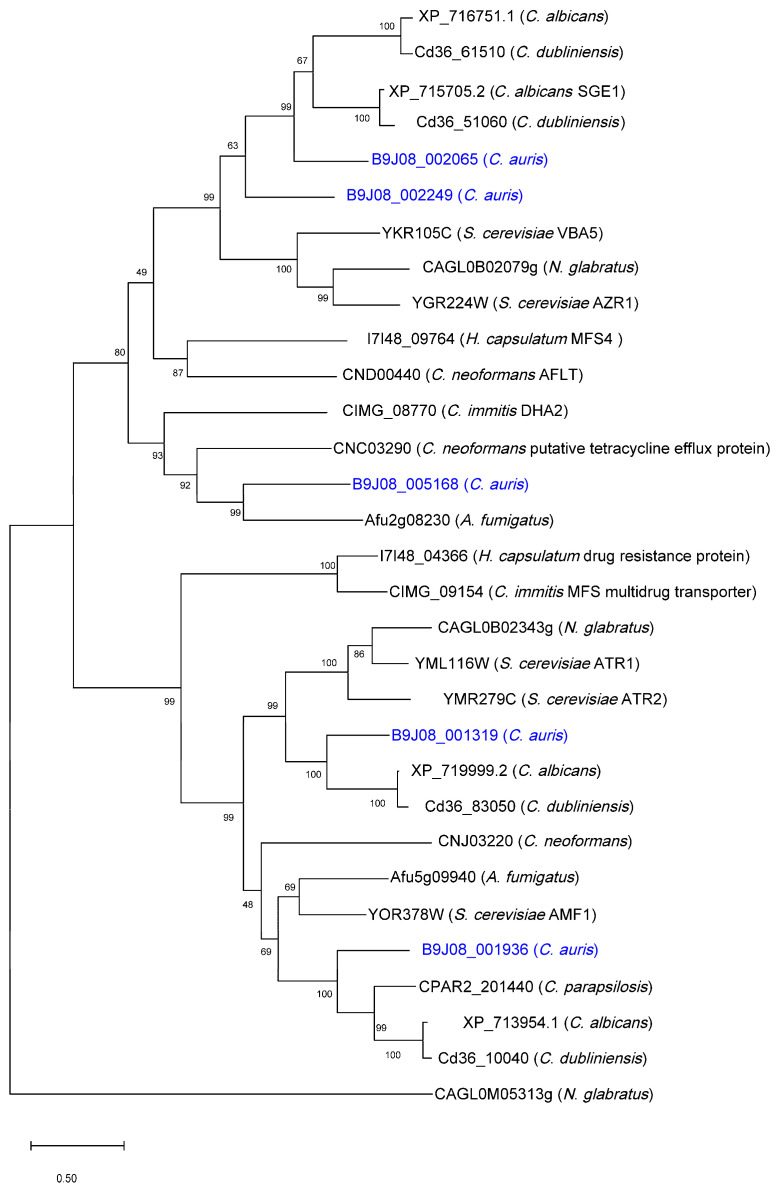
The phylogenetic tree of DHA2 transporters in *C. auris* and other fungal species. The species names include: *Candidozyma (Candida) auris*, *Candida albicans*, *Nakaseomyces (Candida) glabratus*, *Candida dubliniensis*, *Candida parapsilosis*, *Aspergillus fumigatus*, *Cryptococcus neoformans*, *Coccidioides immitis*, *Histoplasma capsulatum*, and *Saccharomyces cerevisiae*. The protein sequences for *C. auris* are highlighted in blue. The phylogeny was inferred using the Maximum Likelihood method and Jones-Taylor-Thornton model [79] of amino acid substitutions and the tree with the highest log likelihood (−32,877.60) is shown. The percentage of replicate trees in which the associated taxa clustered together (1000 replicates) is shown next to the branches [36]. The initial tree for the heuristic search was selected by choosing the tree with the superior log-likelihood between a Neighbor-Joining (NJ) tree [34] and a Maximum Parsimony (MP) tree [96]. Evolutionary analyses were conducted in MEGA12 [33].

Five members of the Drug:H^+^ Antiporter-2 (DHA2) family (TC 2.A.1.3) were identified in *C. auris*. Although their physiological functions remain unknown, these transporters share evolutionary relationships with *DHA2* proteins characterized in other fungi [105], including those in *S. cerevisiae* and *Candida* species (Figure 5). Two putative genes, B9J08_002065 and B9J08_002249, are homologous to the *VBA5* and *AZR1* genes in *S. cerevisiae*, which are involved in drug sensitivity and amino acid uptake [106], and to *SGE1* in *C. albicans*. Two additional genes, B9J08_001319 and B9J08_001936, are paralogous to the low-affinity ammonium transporter *AMF1* (YOR378W) in *S. cerevisiae*. The fifth gene, B9J08_005168, shows sequence similarity to multiple DHA2 transporters from other fungi, including *A. fumigatus*, *C. neoformans*, and *Coccidioides immitis*.

#### 3.2.3. Additional Novel Transporters

The *C. auris* transporter repertoire also includes several systems that have not yet been characterized but may play potential roles in metabolism and stress adaptation. Members of the Vacuolar H^+^-ATPase (V-ATPase) superfamily (TC 3.A.2.2) are involved in maintaining pH balance, metal tolerance, and virulence, as demonstrated in *C. albicans* and *C. glabrata* [107,108,109]. In *S. cerevisiae*, loss of V-ATPase activity results in a conditionally lethal phenotype characterized by sensitivity to extracellular pH [110].

We identified 29 transporters in the Mitochondrial Carrier (MC) Family (TC 2.A.29). MC transporters move metabolites, nucleotides, and cofactors across the mitochondrial membrane to power processes essential for energy production, stress response, and pathogenicity [111,112]. In *S. cerevisiae,* MC transporters are central to metabolic signaling, amino acid biosynthesis, redox shuttling, and energy generation [113]. Notably, disruption of a mitochondrial phosphate carrier *MIR1* in *C. albicans* leads to ATP depletion, accumulation of reactive oxygen species (ROS), and defects in filamentation [114], suggesting that MC transporters are potentially promising antifungal targets [114,115,116].

### 3.3. Transmembrane Topology and Predicted Substrates of C. auris Transporters

**Figure 6:**
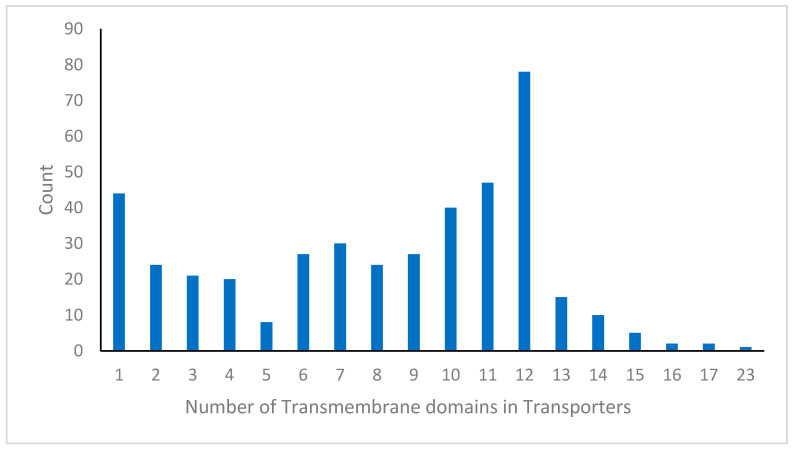
Topological distribution of predicted transporters in *C. auris*. Analysis using TMHMM revealed that 386 transporters in *C. auris* lacked identifiable transmembrane helices.

The number and arrangement of transmembrane segments (TMSs) largely define a transporter’s structural configuration and membrane orientation [117]. To investigate the structural diversity of transporters in *C. auris*, we predicted TMS topology using TMHMM [118]. The TMS counts ranged from none to 23 (Figure 6). In fungi, TMS numbers and organization vary across transporter families and often reflect distinct functional adaptations. For instance, the ABCF transporters in *C. auris* lack transmembrane domains, similar to their counterparts in other species, which are thought to participate in translation and antibiotic resistance [90]. Although the functional implications of these structural differences remain unclear, most predicted transporters were classified as channels or pores, electron carriers, electrochemical potential-driven transporters, or group translocators, consistent with typical fungal membrane transport systems.

**Figure 7:**
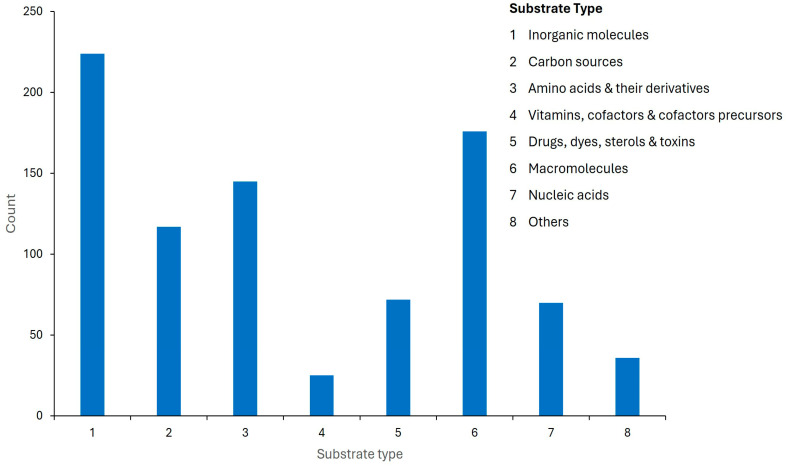
Predicted substrate classes of transporter proteins in the *C. auris* genome. Transporter substrate types were inferred using the ChEBI chemical ontology [30].

The predicted transporters in *C. auris* are associated with a broad range of substrates, including macromolecules, inorganic compounds, carbon sources, amino acids and their derivatives, nucleic acids, drugs, dyes, toxins, and vitamins (Table S2 and Figure 7).

We identified a set of metal-ion transporters in *C. auris* spanning four conserved families that mediate zinc, iron, and copper uptake. *C. auris* contains four members of the Zinc (Zn^2+^)–Iron (Fe^2+^) Permease (ZIP) family (TC 2.A.5), six members of the Iron/Lead Transporter (ILT) family (TC 2.A.108), three members of the Metal Ion (Mn^2+^–iron) Transporter (Nramp) Family (TC 2.A.55), and one member of the Copper Transporter (Ctr) family (TC 1.A.56) (Table 2).

In *S. cerevisiae*, *ZRT1* and *ZRT2* act as high- and low-affinity zinc transporters, respectively, to mediate zinc storage and homeostasis [119,120]. In *C. albicans*, *ZRT1* and *ZRT2* work with the zincophore *PRA1* to support growth under zinc-limited conditions and contribute to virulence [121,122,123,124,125]. The presence of four ZIP transporters in *C. auris* suggests a similar zinc uptake system.

*C. auris* also contains six ILT family members, including homologs of the well-characterized high-affinity iron uptake proteins FTR1 and FET3. In *C. albicans*, the FET3-FTR1 complex mediates reductive iron assimilation under host-imposed iron limitation and is required for growth and virulence [126,127,128]. The ILTs in *C. auris* may support comparable iron acquisition and homeostasis in iron-restricted environments.

Three *NRAMP* transporters were identified in *C. auris*. *NRAMP* proteins are conserved transporters that mediate the uptake of manganese and iron [129]. In *S. cerevisiae*, the homologs *SMF1* and *SMF2* function in Mn^2+^ uptake, and *SMF3* mobilizes vacuolar iron under iron-limiting conditions [130,131]. In *C. albicans*, recent studies show that the *SMF11*, *SMF12*, *SMF13*, and *SMF2/3* transporters contribute to manganese homeostasis, antifungal tolerance, morphogenesis, and pathogenesis [132,133,134].

A single *CTR*-family transporter was identified in *C. auris*. In *C. albicans*, *CTR* proteins mediate high-affinity copper uptake, which is critical under copper-limited conditions [135]. The presence of a *CTR* homolog in *C. auris* indicates that copper acquisition may similarly contribute to its survival and virulence.

### 3.4. Transcriptomic Analysis of Predicted C. auris Transporters Under Antifungal Stress

After compiling a comprehensive set of predicted transporter genes in *C. auris*, we assessed their functional relevance. While transporters are central to antifungal tolerance, stress adaptation, and host–pathogen interactions, sequence-based annotation alone offers limited biological context. We therefore integrated publicly available transcriptomic datasets to identify transporters regulated by antifungal exposure or host-relevant stresses.

To prioritize transporters with potential roles in antifungal tolerance, we focused on three RNA-seq datasets that together capture the major clinical and biological contexts in which transporter activity is expected to be relevant in *C. auris*. These datasets were selected because they collectively represent the three principal classes of antifungal drugs used to treat *C. auris* infections [136,137]. Specifically, Shivarathri et al. (2022) [37] examined transcriptional differences associated with resistance to the polyene amphotericin B, Zamith-Miranda et al. (2021) [38] characterized the transcriptional response to caspofungin in the echinocandin class, and Bing et al. (2020) [39] analyzed gene expression changes linked to fluconazole resistance within the azole class. Together, these studies provide broad coverage of antifungal mechanisms targeting the cell membrane, cell wall, and ergosterol biosynthesis pathways [5,138,139].

These datasets also highlight different temporal and mechanistic aspects of the stress response. The caspofungin data compares treated vs. untreated cells, identifying acute transcriptional shifts and transporters rapidly regulated upon exposure [38]. In contrast, the amphotericin B [37] and fluconazole [137] datasets compare resistant and susceptible isolates, revealing stable expression profiles associated with long-term adaptation.

To avoid confounding technical variation, each RNA-seq dataset was analyzed independently. Because the three datasets represent distinct classes of antifungal drugs with different cellular mechanisms, differential expression analyses were performed separately to evaluate transporter responses in a drug-specific manner. Differentially expressed transporters for each condition are reported in Table S3.

**Figure 8:**
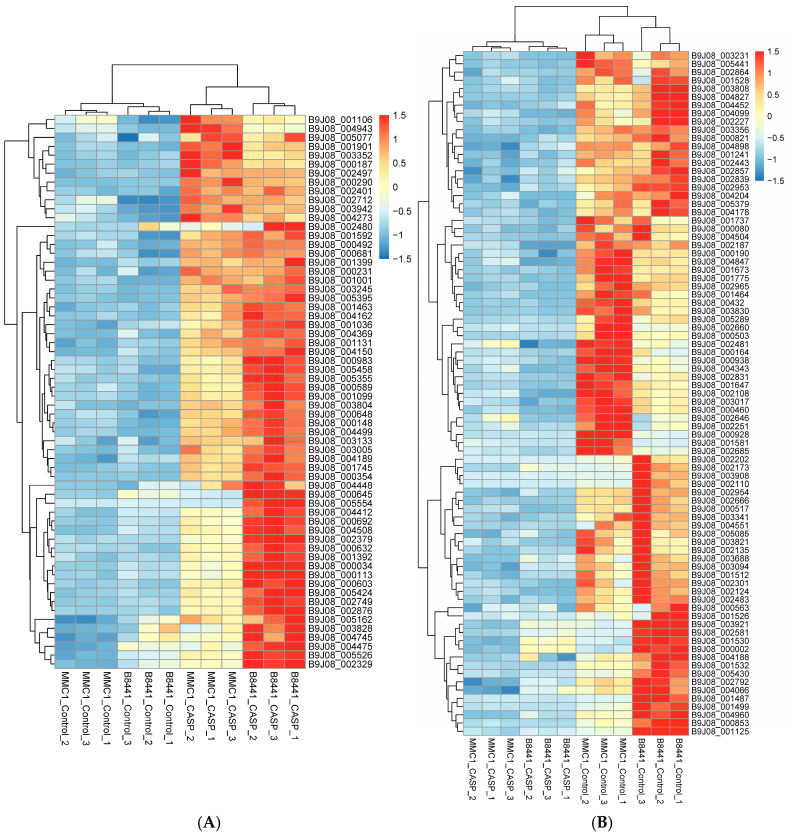
Differential expression patterns of predicted *C. auris* transporter genes in response to caspofungin in the B8441 and MMC1 strains [38]. (**A**) Upregulated transporters. (**B**) Downregulated transporters. Rows represent individual transporter genes (accession numbers), and columns represent individual RNA-seq samples. Expression values were z-transformed by gene (row-wise) to highlight relative expression changes across conditions. Hierarchical clustering was performed on both rows and columns using Euclidean distance and complete linkage. Triplicate samples correspond to the following run accession numbers: SRR13182982 (B8441_Control_1), SRR13182981 (B8441_Control_2), SRR13182970 (B8441_Control_3), SRR13182959 (B8441_CASP_1), SRR13182952 (B8441_CASP_2), SRR13182951 (B8441_CASP_3), SRR13182950 (MCC1_Control_1), SRR13182949 (MCC1_Control_2), SRR13182948 (MCC1_Control_3), SRR13182947 (MCC1_CASP_1), SRR13182980 (MCC1_CASP_2), and SRR13182979 (MCC1_CASP_3).

In response to caspofungin treatment, 325 transporters were differentially expressed in the B8441 strain, including 147 upregulated and 176 downregulated genes, whereas in the MMC1 strain, 112 transporters were upregulated and 125 were downregulated. Both strains shared 62 upregulated transporters (Figure 8A), specifically eight nuclear pore complex (NPC) members: B9J08_001099, B9J08_004162, B9J08_002497, B9J08_000692, B9J08_001463, B9J08_000492, B9J08_001745, and B9J08_004369. This mobilization of the NPC likely accelerates the nucleocytoplasmic trafficking of transcription factors and mRNAs, which are essential for survival under echinocandin-induced stress [140]. Metabolic adaptation was evidenced by the regulation of five MFS transporters (B9J08_002480, B9J08_004448, B9J08_004475, B9J08_005162, and B9J08_003352) from the Sugar Porter and Siderophore–Iron (SIT) families. Caspofungin also triggered a shift in mitochondrial activities. Three mitochondrial carrier proteins (B9J08_000034, B9J08_000231, B9J08_001399) and three mitochondrial protein translocase (MPT) components (B9J08_001392, B9J08_004412, B9J08_003133) were upregulated. Increased metabolite flux and the import of nuclear-encoded proteins suggest the cell’s compensation for the high energetic cost of restoring cell wall integrity. In contrast, 83 predicted transporters were downregulated in both strains under caspofungin stress (Figure 8B). These genes span multiple transporter families, including 21 members in the MFS, eight members in the Amino Acid-Polyamine-Organocation (APC) Superfamily, and six members in the ABC superfamily, consistent with broad transcriptional reprogramming during caspofungin exposure, alongside previously reported alterations in cell wall, ribosomal, and cell cycle-associated functions [38].

In the case of AmB, we observed complex isolate-specific expression patterns. Compared with the susceptible isolate, 33 transporters were differentially expressed in AmBR1 (4 upregulated and 29 downregulated), 84 in AmBR2 (33 upregulated and 51 downregulated), and 36 in AmBR3 (13 upregulated and 23 downregulated) (Table S3). Several drug efflux transporters were induced in at least one AmB-resistant isolate, including the MFS transporters B9J08_004662 (*HOL4*), B9J08_004040 (*HOL1*), B9J08_004775 (*TPO3*), and B9J08_004113 (*C. albicans MDR1* homolog), as well as the ABC transporter B9J08_000479 (*CDR4*). In addition, multiple predicted hexose transporters (*HGT2*, *HGT10*, *HGT12*, *HGT17*, and *HGT19*) and *SIT1* were upregulated, indicating broader metabolic reprogramming during antifungal stress. Their *C. albicans* homologs, *HGT12* and *HGT7*, are regulated by the glucose sensor *HGT4* and contribute to virulence and metabolic adaptation [141,142,143].

Fluconazole resistance in *Candida* species has been studied extensively. In *C. albicans*, resistance is driven by a combination of mutations in the ergosterol biosynthesis pathway and altered regulation of drug-response genes, including transcription factors such as *TAC1*, which influences downstream gene expression and cellular adaptation to azoles [144,145]. In *C. auris*, mutations in the zinc cluster transcription factor *TAC1B* have been identified in multiple clinical isolates and confer increased fluconazole resistance; these mutations also elevate expression of effector genes involved in drug response [76,77]. Our reanalysis of fluconazole RNA-seq data identified 51 upregulated transporters and 65 downregulated transporters (Table S3). Both *TAC1A* and *TAC1B* were upregulated, accompanied by increased expression of *CDR1* (B9J08_000164) and *MDR1* (B9J08_004113) by 1.5-fold and 4.2-fold, respectively, in the fluconazole-resistant strain. These observations are consistent with the recent study by Barker et al. [76], which demonstrated that mutations in *TAC1B* drive resistance to fluconazole and broader triazole antifungals by upregulating *CDR1* and *MDR1*. In addition to *CDR1* and *MDR1*, several MFS genes, including *HGT7*, *SIT1*, *SIT1*, *HOL4*, and *TPO3* appeared to be upregulated.

### 3.5. Phylogenetic Context and Lineage-Specific Divergence in Predicted C. auris Transporters

Before we investigated the transporter divergence within an evolutionary framework, we reconstructed the phylogeny of representative Saccharomycotina species using ITS sequences (Figure S1). Our results align with recent phylogenomic analyses [17], verifying that *C. auris* occupies a distinct lineage within the Saccharomycotina, divergent from both *S. cerevisiae* and other *Candida* pathogens.

Species selection for comparative transporter analysis was guided by clinical significance and genomic data quality, with curated orthology data available in the *Candida* Genome Database. We included *S. cerevisiae* as a functional anchor to leverage its experimentally validated proteome. This selection strategy ensures the analysis is grounded in high-confidence functional data while capturing the evolutionary diversity across both pathogenic and non-pathogenic lineages.

Within this framework, we assessed lineage-specific divergence of predicted transporter families in *C. auris* relative to *S. cerevisiae*, *C. albicans*, *C. dubliniensis*, *N. glabrata*, and *C. parapsilosis* (Table S4 and Table 3).

The *C. auris* genome contains 48 predicted Nuclear Pore Complex (NPC) components (Table 3), an intermediate repertoire compared to the 63 in *S. cerevisiae* and the 31–34 found in *C. albicans*, *C. dubliniensis*, and *C. parapsilosis*. As NPC proteins are dosage-sensitive regulators of nucleocytoplasmic trafficking and stress-responsive transcription [146,147], their induction during caspofungin exposure may facilitate the rapid adaptive reprogramming required to survive echinocandin stress.

The MFS is expanded in *C. auris* (96 members) relative to *C. albicans* (64), *C. dubliniensis* (59), and *N. glabrata* (47), and comparable to *C. parapsilosis* (93) and *S. cerevisiae* (82). The expansion of DHA1 and DHA2 family transporters in *C. auris* suggests an evolutionary investment in transporter-mediated stress adaptation. These transporters have broad substrate specificity and are important for multidrug tolerance, pH homeostasis, and detoxification [14,97,105].

*C. auris* maintains a higher copy number of siderophore–iron transporter (SIT) family members than other pathogenic *Candida* species and *S. cerevisiae*. Multiple SITs were differentially expressed across multiple datasets, including those involving caspofungin treatment and isolates resistant to amphotericin B and fluconazole. Given that SITs are essential for fungal iron acquisition and stress adaptation, and that iron homeostasis frequently modulates drug susceptibility in *Candida* species [148,149,150,151], these genes may be central to *C. auris* fitness under drug pressure. The overlap of lineage-specific gene expansion and responsive transcriptional regulation suggests that SIT transporters are a dynamically regulated element of the *C. auris* stress response [21,38,152].

The ABC superfamily comprises 26 members in *C. auris*, compared with 22 in *C. albicans*, 21 in *C. dubliniensis*, 22 in *N. glabrata*, 30 in *C. parapsilosis*, and 28 in *S. cerevisiae*, indicating overall conservation across species.

Taken together, transporter family representation in *C. auris* reflects both conserved Saccharomycotina-wide features and lineage-associated differences. The inclusion of *S. cerevisiae* as a reference species allows comparison to a well-characterized functional baseline, while the pathogenic *Candida* species provide a clinical context.

## 4. Discussion

In this study, we conducted a comprehensive genomic, evolutionary, and transcriptomic analysis of membrane transport systems in *C. auris*, a multidrug-resistant fungal pathogen of global concern. By integrating TCDB-based annotation, phylogenetic inference, comparative genomics, and reanalysis of multiple transcriptomic datasets, we provide the first systematic framework for understanding the components, regulation, and potential functional relevance of transporters in *C. auris*. Rather than serving as a static catalog, this analysis reveals transporter diversification and expression that point to strategies underlying antifungal tolerance, stress adaptation, and pathogenicity.

Transporter annotation in this study was not based on percent sequence similarity alone, which can be misleading for membrane proteins due to conserved transmembrane architecture. Instead, we applied a combination of stringent BLAST E-value thresholds (≤10^−30^), alignment coverage requirements, domain architecture validation, and phylogenetic analysis to support transporter assignments. Furthermore, the TCDB includes both core transporters and accessory factors involved in transport, reflecting a systems-biology perspective in which transport activity is mediated by protein complexes, chaperones, and regulatory cofactors.

Class 2 electrochemical potential-driven transporters, particularly the MFS, are enriched. Expansion of these transporter families may be an adaptive outcome to the high-stress conditions of healthcare environments, including nutrient limitation, host-imposed stresses, and antifungal exposure. The diversification of transporters allows *C. auris* to fine-tune substrate uptake, detoxification, and intracellular trafficking in response to environmental challenges.

Efflux-mediated antifungal resistance is one of the best-characterized functions of transporters in pathogenic fungi [58,153,154]. Our identification of the expanded MFS and ABC superfamily in *C. auris* reinforces their importance. However, recent mechanistic advances demonstrate that resistance phenotypes are not driven solely by transporter copy number, but by regulatory rewiring that changes transporter expression. In *C. auris*, gain-of-function mutations in the zinc cluster transcription factor *TAC1B* have been shown to be a pivotal mechanism driving clinical azole resistance by upregulating *CDR1* and *MDR1* [76,155]. Consistent with this regulatory model, our reanalysis of fluconazole transcriptomic data revealed increased expression of *TAC1A* and *TAC1B*, along with elevated expression of *CDR1* and *MDR1*, in resistant isolates. These data link genomic content to resistance regulation and place efflux pumps within a transcriptional network, rather than treating them as isolated resistance determinants. Our data suggest that *TAC1B*-associated regulatory changes may extend beyond efflux pumps. In addition, several MFS transporters involved in sugar uptake and siderophore-mediated iron acquisition were also differentially expressed in resistant isolates. This observation suggests that *TAC1B* and related regulators may coordinate a metabolic and stress-adaptation program that couples drug efflux with nutrient utilization and ion homeostasis. Direct testing of this hypothesis will require genome-wide mapping of *TAC1B* binding sites and functional characterization of downstream transporter targets.

We conducted comparative analyses that incorporated both clinically relevant *Candida* species and the model yeast *S. cerevisiae*. The inclusion of *S. cerevisiae* provided a functional baseline for conserved transporter families, while comparisons with pathogenic *Candida* species could reveal features potentially associated with clinical adaptation. Beyond efflux systems, our analysis highlights several transporter families whose expansion and regulation suggest underexplored roles in *C. auris* biology. The siderophore–iron transporter (SIT) family is among the most expanded groups. *C. auris* encodes more SIT homologs than other *Candida* species. Several SITs were differentially expressed in response to caspofungin, amphotericin B, and fluconazole exposure. Iron acquisition is associated with fungal virulence, oxidative stress response, and antifungal susceptibility [149,152]. The lineage-specific expansion and antifungal-responsive expression suggest that SIT transporters contribute to *C. auris* fitness under iron-limited and drug-stressed conditions.

Our genomic analysis also identified an expansion of nuclear pore complex (NPC) components in *C. auris*. NPC proteins are not only static gates, but also active regulators of transcription, mRNA export, and signaling [60,147,156]. The transcriptional induction of NPC components during caspofungin treatment suggests that *C. auris* may enhance nucleocytoplasmic trafficking to support rapid transcriptional reprogramming in response to cell wall stress. Efficient transport of stress-response regulators may confer a selective advantage under echinocandin pressure.

Transporter systems such as the vacuolar H^+^-ATPase (V-ATPase) and mitochondrial carrier (MC) families are important but remain poorly characterized in *C. auris*. In *C. albicans* and *N. glabrata*, genetic disruption of V-ATPase function causes vacuolar alkalinization, impaired filament production, and increased sensitivity to antifungal stresses, suggesting their importance for fungal fitness in the host environment [109,157,158,159]. V-ATPase activity is also associated with virulence, as ergosterol is essential for V-ATPase assembly; fluconazole-mediated inhibition of ergosterol biosynthesis directly impairs vacuolar acidification [108]. Furthermore, the presence of 29 MC transporters in *C. auris* suggests a high capacity for metabolic exchange during nutrient limitation, oxidative stress, or antifungal exposure. Targeted functional studies of individual MCs in *C. auris*, for example, assessing respiratory efficiency, ROS dynamics, and virulence phenotypes, will help determine whether these transporters represent potential targets for interventions.

The persistence of *C. auris* in clinical environments and its ability to survive antifungal treatment is an integrated phenotype involving cell wall properties, metabolic flexibility, and transcriptional regulation [160,161,162]. Structural analyses have demonstrated that the *C. auris* cell wall is distinct from that of other pathogenic *Candida* species in both composition and antifungal response [160], suggesting coordination between transporter activity and cell wall remodeling under drug pressure. Metabolic plasticity further contributes to the fitness of *C. auris*: pan-drug-resistant *C. auris* isolates have shown altered utilization of carbon and nitrogen sources without a deleterious fitness effect, indicating that metabolic reprogramming is associated with antifungal resistance [161]. These traits are consistent with the expansion and regulation of transporter families identified in this study, particularly those involved in nutrient uptake, ion balance, and mitochondrial metabolism. At the regulatory level, transcription factors such as *TAC1B*, are central to this resilience phenotype [76,77,155]. The regulatory plasticity and lineage-specific expansion of transporter families support that drug efflux, metabolism, and stress responses are coordinated through shared regulatory circuits.

While this study provides a comprehensive overview of transporter composition, expansion, and transcriptional responses, further work is needed to elucidate the regulatory mechanisms. Promoter analysis for conserved transcription factor binding motifs could generate testable hypotheses for how transporter expression is coordinated under stress or drug exposure. In *C. albicans*, transcription factors such as *TAC1*, *MRR1*, *CAP1*, and *ZAP1* have been shown to control multidrug efflux, oxidative stress responses, and metal homeostasis via binding to specific promoter motifs [163,164,165,166]. Similar analyses in *C. auris* [76,77], coupled with experimental validation using approaches such as ChIP-seq, would help reveal genome-wide regulatory networks linking transporter expansion to adaptive phenotypes.

Despite these insights, a limitation of this study is the reliance on homology-driven annotation. While sequence similarity and TCDB classification provide a baseline for cataloging transport systems, these methods cannot resolve precise substrate specificity, regulatory logic, or physiological impact. Functional divergence among homologs is common, particularly within lineage-specific families. Additionally, while transcriptomic analyses identify condition-dependent expression, they do not establish causality. Therefore, the annotations and inferences presented here serve as a framework for hypothesis generation rather than a definitive functional map.

The datasets presented in this study provide a roadmap for prioritized functional studies. High-priority candidates for validation include those transporter families that exhibit both lineage-specific expansion and antifungal-responsive expression. Direct evidence for their role in resistance and stress tolerance needs to be established through systematic genetic disruption and subsequent fitness assays. Furthermore, the underlying regulatory logic remains to be defined. Defining the *TAC1B* cistrome in parallel with transcriptomic profiling of specific regulatory mutants will distinguish direct TAC1B targets from those in broader, indirect stress-response circuits. Moving beyond these genomic observations toward a holistic model of *C. auris* resilience requires experimental frameworks that bridge transporter function with cell wall and metabolic dynamics. Such an approach is necessary to resolve how resistance emerges from coordinated, network-level adaptations rather than isolated genetic events.

In summary, the expanded and coordinated transporter repertoire of *C. auris* is central to its environmental resilience and stress tolerance. Transitioning from genomic cataloging to functional characterization of these systems is critical for establishing their mechanistic roles and determining their potential as therapeutic targets.

## Figures and Tables

**Table 1:** Distribution of predicted transporters in the *C. auris* B8441 genome based on the Transporter Classification (TC) system. Two-letter prefixes (VW) indicate the respective TC class and subclass.

Class	Subclass	*C. auris*
**1: Channels/Pores**		**115 (14%)**
	1.A: α-Type Channels	41
	1.B: β-Barrel Porins	4
	1.C: Pore-Forming Toxins (Proteins and Peptides)	2
	1.F: Vesicle Fusion Pores	8
	1.H: Paracellular Channels	2
	1.I: Membrane-bounded Channels	48
	1.N: Cell Fusion Pores	4
	1.P: Non-Envelop Virus Penitration Complex: A complex of host cell proteins that allow non-envelop virus to penetrate the endoplasmic reticular membrane.	3
	1.R: Membrane Contact Site for Interorganellar Transport	3
**2: Electrochemical Potential-driven Transporters**		**268 (33%)**
	2.A: Porters (uniporters, symporters, antiporters)	264
	2.D: Transcompartment Lipid Carrier	4
**3: Primary Active Transporters**		**195 (24%)**
	3.A: P-P-bond-hydrolysis-driven transporters	167
	3.B: Decarboxylation-driven transporters	1
	3.D: Oxidoreduction-driven transporters	27
**4: Group Translocators**		**14 (2%)**
	4.C: Acyl CoA ligase-coupled transporters	6
	4.D: Polysaccharide Synthase/Exporters	5
	4.E: Vacuolar Polyphosphate Polymerase-catalyzed Group Translocators	2
	4.F: Choline/EthanolaminePhosphotransferase 1	1
**5: Transmembrane Electron Carriers**		**7 (1%)**
	5.B: Transmembrane 1-electron transfer carriers	7
**8: Accessory Factors Involved in Transport**		**125 (15%)**
	8.A: Auxiliary transport proteins	125
**9: Incompletely Characterized Transport Systems**		**87 (11%)**
	9.A: Recognized transporters of unknown biochemical mechanism	35
	9.B: Putative transport proteins	52
**Total**		**811**

**Table 2:** Representative transporters involved in metal ion transport in *C. auris*.

Transporter TC	Accession Number	Homologous Sequence in *C. albicans*	
		Accession Number	Gene Name
2.A.5: The Zinc (Zn^2+^)–Iron (Fe^2+^) Permease (ZIP) Family	B9J08_003341	C2_02590W	*ZRT2*
	B9J08_000003	C4_06970C	*ZRT1*
	B9J08_002992	C4_06980W	*PRA1*
	B9J08_003657	C2_02590W	*ZRT2*
2.A.108: The Iron/Lead Transporter (ILT) Family	B9J08_000517	C6_00480C	*FET31*
	B9J08_002997	C5_00460C	*FET3*
	B9J08_002108	C1_14130W	*FTR1*
	B9J08_002464	CR_01270C	*FTH2*
	B9J08_003002	C1_14130W	*FTR1*
	B9J08_000170	C1_09400C	*FTH1*
2.A.55: The Metal Ion (Mn^2+^–iron) Transporter (Nramp) Family	B9J08_002789	C2_07160W	*SMF12*
	B9J08_003040	C1_13840W	*SMF13*
	B9J08_002199	C2_00580C	*SMF3*
1.A.56: The Copper Transporter (Ctr) Family	B9J08_000258	C1_08620W	*CTR2*

**Table 3:** Comparative distribution of representative transporter families in *C. auris* and selected Saccharomycotina species.

Transporter Superfamily/Family		Common Family Name	*C. auris*	*C. albicans*	*C. dubliniensis*	*N. glabrata*	*C. parapsilosis*	*S. cerevisiae*
1.I.1 The Nuclear Pore Complex (NPC) Family		NPC	**48**	**31**	**30**	**45**	**31**	**63**
2.A.1 The Major Facilitator Superfamily (MFS)		MFS	**96**	**64**	**59**	**47**	**93**	**82**
	2.A.1.1: The Sugar Porter (SP) Family	SP	*25*	*15*	*15*	*17*	*18*	*31*
	2.A.1.2: The Drug:H+ Antiporter-1 (12 Spanner) (DHA1) Family	DHA1	*15*	*13*	*11*	*10*	*24*	*12*
	2.A.1.3: The Drug:H+ Antiporter-2 (14 Spanner) (DHA2) Family	DHA2	*5*	*4*	*4*	*3*	*1*	*7*
	2.A.1.9: The Phosphate: H+ Symporter (PHS) Family	PHS	*4*	*2*	*2*	*1*	*4*	*2*
	2.A.1.14: The Anion:Cation Symporter (ACS) Family	ACS	*17*	*13*	*10*	*5*	*19*	*10*
	2.A.1.16: The Siderophore-Iron Transporter (SIT) Family	SIT	*16*	*1*	*1*	*1*	*5*	*6*
2.A.7 The Drug/Metabolite Transporter (DMT) Superfamily		DMT	**9**	**5**	**6**	**5**	**5**	**10**
2.A.18: The Amino Acid/Auxin Permease (AAAP) Family		AAAP	**9**	**5**	**7**	**7**	**3**	**7**
	2.A.18.4: Aromatic and neutral amino acid permease	AAP	*3*	*0*	*0*	*0*	*0*	*0*
2.A.67 The Oligopeptide Transporter (OPT) Family		OPT	**10**	**6**	**7**	**1**	**8**	**3**
3.A.1: The ATP-binding Cassette (ABC) Superfamily		ABC	**26**	**22**	**21**	**22**	**30**	**28**
5.B.1: The Phagocyte (gp91phox) NADPH Oxidase Family		Phox	**7**	**0**	**0**	**0**	**0**	**5**

## Data Availability

All sequence accession numbers are provided within the manuscript.

## Supplementary Materials

The following supporting information can be downloaded at: https://www.mdpi.com/article/10.3390/jof12030174/s1. Table S1: Predicted transporters identified in *C. auris*; Table S2: Distribution of substrate types of predicted *C. auris* transporters; Table S3: Predicted transporters in *C. auris* that are differentially expressed in drug-resistant isolates or under drug-exposed conditions; Table S4: Transporters identified in *S. cerevisiae*, *C. auris*, *C. albicans*, *C. dubliniensis*, *N. glabrata*, and *C. parapsilosis*; Figure S1: Phylogenetic relationships of representative species within the subphylum Saccharomycotina. Species selected for the comparative analysis of the transporter repertoire are marked in blue. The phylogeny was inferred using the Maximum Likelihood method and Tamura-Nei (1993) model [167] of nucleotide substitutions and the tree with the highest log likelihood (−39,868.52) is shown. The percentage of replicate trees in which the associated taxa clustered together (1000 replicates) is shown next to the branches [36]. The initial tree for the heuristic search was selected by choosing the tree with the superior log-likelihood between a Neighbor-Joining (NJ) tree [34] and a Maximum Parsimony (MP) tree [96]. Evolutionary analyses were conducted in MEGA12 [33].

## Abbreviations

The following abbreviations are used in this manuscript:

ABC	ATP-binding cassette
AmB	amphotericin B
CASP	Caspofungin
ChEBI	Chemical Entities of Biological Interest
CTR	Copper Transporter
DCT	Drug Conjugate Transporter
DE	differentially expressed
DHA	Drug:H+ Antiporter
FDR	False discovery rate
FLZ	fluconazole
GEO	Gene Expression Omnibus
GLM	Generalized Linear Model
HMT	Heavy Metal Transporter
ILT	Iron/Lead Transporter
MC	Mitochondrial Carrier
MDR	multidrug resistance
MFS	major facilitator superfamily
MIC	Minimum Inhibitory Concentration
MP	Maximum Parsimony
MPE	Mitochondrial Peptide Exporter
NBD	nucleotide-binding domain
NJ	Neighbor-Joining
NPC	nuclear pore complex
ORF	open reading frame
PDR	Pleiotropic Drug Resistance
ROS	reactive oxygen species
SIT	Siderophore–Iron Transporter
SP	Sugar Porter
STE	Sex Pheromone Exporter
TC	Transport Classification
TCDB	Transporter classification database
TMD	transmembrane domain
TMS	transmembrane segment
TPO	transporter of polyamines
ZIP	Zinc–Iron Permease
