# Supplementary material for: Genomic Analysis Reveals Diversified and Stress-Responsive Transport Repertoire in Candidozyma (Candida) auris
Source: J Fungi (Basel). 2026 Feb 28;12(3):174. doi: 10.3390/jof12030174 (PMC13028451; doi:10.3390/jof12030174)
Supplement: Supplementary file 1 [file jof-12-00174-s001.zip › FigureS1.pdf]

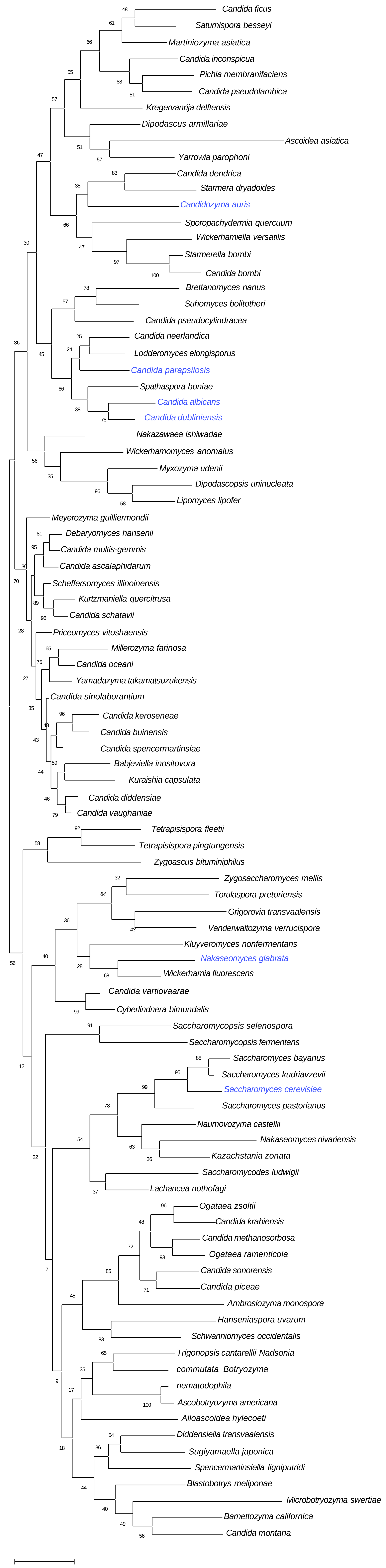

Figure S1: Phylogenetic relationships of representative species within the subphylum Saccharomycotina. Species selected for the comparative analysis of the transporter repertoire are marked in blue. The phylogeny was inferred using the Maximum Likelihood method and Tamura-Nei (1993) model [167] of nucleotide substitutions and the tree with the highest log likelihood (−39,868.52) is shown. The percentage of replicate trees in which the associated taxa clustered together (1000 replicates) is shown next to the branches [36]. The initial tree for the heuristic search was selected by choosing the tree with the superior log-likelihood between a Neighbor-Joining (NJ) tree [34] and a Maximum Parsimony (MP) tree [96]. Evolutionary analyses were conducted in MEGA12 [33].
