# Supplementary material for: Genomic Analysis Reveals Diversified and Stress-Responsive Transport Repertoire in Candidozyma (Candida) auris
Source: J Fungi (Basel). 2026 Feb 28;12(3):174. doi: 10.3390/jof12030174 (PMC13028451; doi:10.3390/jof12030174)
Supplement: Supplementary file 1 [file jof-12-00174-s001.zip › Table S2_substrates.pdf]

Supplementary Table S2. Distribution of substrate types of predicted *C. auris* transporters. Transporters lacking substrate annotations were not included.

Substrate	No. of transporters (class) acting on substrate type							Total
	1. Channels & Pores	2. Primary Carriers	3. Secondary Carriers	4. Group translocators	5. Trans-membrane electron flow carriers	8. Auxiliary proteins	9. (Putative) Poorly characterized	
1. Inorganic molecules (224)								
A. Cation	11	4	0	0	0	0	0	15
B. Anion	24	90	55	0	0	0	2	171
C. electrons	3	25	3	2	0	0	0	33
D. other	0	1	0	0	4	0	0	5
2. Carbon sources (117)								
A. Sugars & polyols	0	36	0	4	0	0	0	40
B. Mono-carboxylates	0	23	2	5	0	2	5	37
C. Di- & tricarboxylates	0	27	3	0	0	0	0	30
D. Organoanion	0	3	0	0	0	0	0	3
E. Aromatic compounds	0	6	1	0	0	0	0	7
3. Amino acids & their derivatives (145)								
A. Amino acids	0	78	0	0	0	0	0	78
B. Amines, amides, and organocations	0	16	4	1	0	0	0	21
C. Peptides	0	38	7	0	0	1	0	46
4. Vitamins and cofactors (25)								
A. Vitamins	0	13	0	0	0	0	0	13
B. Cofactors	0	8	0	0	3	1	0	12
5. Drugs, dyes, sterols & toxins (72)								
A. Multiple drugs	0	1	4	0	0	0	1	5
B. Specific drugs	0	27	25	0	0	0	0	52
C. Pigments & dyes	0	0	8	0	0	0	0	8
D. Sterols	0	0	6	0	0	0	1	7
6. Macromolecules (176)								
A. Carbohydrates	0	0	0	1	0	0	0	1
B. Proteins	8	1	89	0	0	12	15	125
C. Lipids	3	10	14	0	0	3	14	44
D. other macromolecules	0	6	0	0	0	0	0	6
7. Nucleic acids (70)								
A. Nucleic acids	6	33	10	0	0	7	2	58
B. Nucleic acid derivatives	0	10	0	0	0	0	2	12
8. Others (36)								
	14	9	11	0	0	2	0	36
